# Assessment of LD‐V1 radiochromic film low‐dose performance for mega‐voltage radiotherapy quality assurance

**DOI:** 10.1002/acm2.70578

**Published:** 2026-04-09

**Authors:** Ruiming C. Edmondson, Darwin A. Garcia, Victor Malkov, James A. Kavanaugh, John J. Lucido

**Affiliations:** ^1^ Department of Radiation Oncology Mayo Clinic Rochester Minnesota USA

**Keywords:** intensity modulated radiation therapy, quality assurance, radiochromic film

## Abstract

**Background:**

Highly modulated radiotherapy delivery demands functional multileaf collimator (MLC) quality assurance (QA) with the ability to measure very low doses during short (<1 s) temporal segments of beam delivery. A radiochromic film with adequate sensitivity in the few‐cGy range is therefore needed. LD‐V1 film was designed for 2–20 cGy measurements in diagnostic kV energy ranges, but its performance for megavoltage (MV) beam qualities has not been well characterized.

**Purpose:**

To demonstrate the feasibility of LD‐V1 film for low‐dose MV measurements and characterize its signal dependence under conditions relevant for clinical QA.

**Methods:**

A 6 MV calibration curve was generated using eight dose levels between 0 and 40 cGy and compared with EBT4 film irradiated under identical conditions. Film characteristics evaluated included dose response, scanner and film signal uniformity, scan repeatability, energy dependence, post‐exposure signal evolution, scanning‐orientation dependence, and one‐dimensional (1‐D) profile visualization.

**Results:**

LD‐V1 demonstrated higher net optical density (OD) and significantly less measurement uncertainty compared to EBT4 at dose levels less than 40 cGy. Significant energy dependence between kV and MV beam qualities was seen. Average net OD differences of 6.3% were seen between landscape and portrait scanning orientations. Post‐irradiation signal growth followed a logarithmic trend, stabilizing at ∼24 h. Scan‐to‐scan variation was <0.1%. Film‐signal uniformity was within 3.9%. 1‐D profiles showed discernible dose fall‐off for doses as low as 10 cGy.

**Conclusions:**

LD‐V1 demonstrates suitability for low‐dose MV measurements and exhibits improved low‐dose sensitivity relative to EBT4, indicating its potential value for MLC QA in highly dynamic treatment deliveries. Nonetheless, its pronounced energy and scanning‐orientation dependence underscores the need for rigorous and consistent calibration and handling procedures.

## 1 INTRODUCTION

Radiation therapy patients can benefit from both improvements in treatment plan quality and reduced treatment delivery times. These improvements include the growing adoption of treatment modalities such as stereotactic radiosurgery,^1^ stereotactic body radiotherapy,^2^ and volumetric‐modulated radiotherapy for craniospinal axis irradiation,^3^ which demand complex and highly‐modulated treatment plans. To support the growing complexities of treatment modalities and dosimetric demands, medical linear accelerator manufacturers are developing new technologies capable of delivering more complex and modulated treatment plans. Examples include double‐stacked multi‐leaf collimators (MLCs),^4^, ^5^ novel gantry‐rotation dynamics,^6^ and the use of new optimization strategies that simultaneously increase modulation and allow the introduction of collimator angle rotation during beam delivery.^7^ In parallel with the interest in reducing beam‐on time, this increased plan complexity results in demanding trajectories for the MLCs (and other components), with large accelerations and velocities.

Ensuring that these complicated treatment plans are accurately delivered remains a critical part of clinical practice. This requires resolving the performance of individual MLC leaves during the short time intervals when these stresses are maximized. Ultimately, these dynamics occur in time frames of a second or less, which corresponds to relatively low absolute doses: even at the highest available dose rates, dosimeters must be able to reliably measure dose differences for irradiations of around 10 cGy.

Radiochromic film provides a powerful tool for high‐resolution two‐dimensional dosimetric measurements,^8^ and is routinely used for both machine quality assurance (QA) (beam profiles^9^ and MLC characterization^10^) and patient‐specific intensity modulated radiotherapy (IMRT) QA^11^ for megavoltage (MV) treatment beams. They are capable of higher spatial resolution than planar diode arrays, and do not suffer the time‐averaging of on‐board electronic portal imagers. However, the radiochromic films typically used for MV applications, such as EBT4 films (Ashland, Bridgewater, NJ), have minimum recommended doses ranging from 20 cGy^12^ to more than 1000 cGy.^8^ Alternatively, radiochromic films intended for diagnostic radiology QA are designed to be used in these low‐dose regimes. In particular, GafChromic LD‐V1 film (Ashland, Bridgewater, NJ) is developed to have a dose range of 2–20 cGy, and an energy range of 40–160 kV. The film characterization and dosimetric accuracy of LD‐V1 films have been established for doses as low as 5 cGy for various kV energies, including mammography beam qualities (24–40 kV),^13^, ^14^, ^15^ orthovoltage unit beam qualities (60–180 kV),^16^ and diagnostic x‐ray beam qualities (90–120 kV).^17^ Because LD‐V1 incorporates materials with higher effective atomic number relative to EBT4 films, its response under MV irradiation may deviate from near tissue‐equivalent behavior, necessitating thorough characterization and validation before clinical use.^8^ This study aims to explore low‐dose sensitivity of LD‐V1 film in an MV beam, characterize its performance in MV beam QA applications, and report the methods required for film handling and analysis.

## 2 METHODS

### 2.1 Dose response

All films used in this study were from the same production lot (lot number 02142403). A single 20 cm × 25 cm LD‐V1 Gafchromic film was cut into 2.5 cm × 12.5 cm strips. Films were irradiated using a TrueBeam linear accelerator (Varian Medical Systems, Palo Alto, CA) calibrated for absolute dosimetry according to AAPM Task Group 51 (TG‐51) protocol using an ADCL‐calibrated Farmer‐type ionization chamber. Films were irradiated at 100 cm source‐to‐surface distance (SSD), 10 cm × 10 cm field, 5 cm depth in solid water with 10 cm thick solid water distal to the films to assure full backscatter. All film strips were scanned in a single row along the center of a flatbed scanner (Epson Expression 13000XL, Suwa, Japan) in landscape orientation, using reflective mode, 200 dpi, 48‐bit RGB channel. A centered 1.5 cm × 3.0 cm region of interest (ROI) was drawn on each film strip for data collection.

A calibration curve was built using red channel net optical density (OD) of the 6 MV beam at 46 h post‐irradiation with color reciprocal linear vs dose fitting model (FilmQA Pro software, v.7) using eight dose levels of 0.00, 5.10, 10.4, 15.9, 21.6, 27.5, 33.6, and 40.0 cGy. Net OD was calculated as the OD difference between the unirradiated control and the irradiated film. To assess reproducibility, an independent set of strips from the same film box was irradiated using the same dose levels, and two additional dose levels (20 and 25 cGy) were included using film from a different box within the same production lot.

Additionally, to compare the low‐dose response of LD‐V1 with that of EBT4, an EBT4 calibration curve was generated using film strips of identical size, irradiated at the same eight dose levels under 6 MV beam quality, following the same experimental setup used for LD‐V1.

### 2.2 Post‐irradiation time, scanning direction, and energy dependence

All post‐irradiation scans were performed using the same film alignment, scanning parameter, and ROI selection as the dose‐response characterization. The 6 MV film strips were scanned at 1.5, 3, 6, 12, 24, and 46 h post‐irradiation. Dose measurement errors were assessed by applying the 46 h post‐irradiation calibration curve to each of the earlier scans, using the one‐scan protocol of FilmQA Pro.^18^ Scanning‐direction dependence was evaluated by rescanning the 6 MV film strips in portrait orientation (rotated 90°) and comparing the resulting net OD values with those obtained in the original landscape orientation.

To assess energy dependence, film strips of same size from the same box were used for irradiation with 10 and 18 MV beams of the same eight dose levels as used for dose‐response characterization. For comparison against kV responses, additional films were exposed using an Xstrahl 300 orthovoltage unit (Xstrahl Inc., Sugar Hill, GA) calibrated for absolute dosimetry according to AAPM Task Group 61 using an ADCL‐calibrated Farmer‐type ionization chamber.^19^ The same eight dose levels (rounded to the nearest MU) of 0, 5, 10, 16, 22, 28, 34, and 40 cGy were used. The films were irradiated at 1 cm depth in a 30 cm × 30 cm × 10 cm solid water phantom with a 10 cm diameter cone, 30 cm focus‐to‐source distance, at 100, 180, and 250 kV (half‐value layers of 3.0 mm Al, 0.5 mm Cu, and 2.0 mm Cu, respectively).

### 2.3 One‐dimensional (1‐D) dose profile visualization

For the 6 MV and 100 kV LD‐V1 film strips, 1‐D absolute and relative dose profiles were generated by extracting line profiles through the center of each film along the x‐axis, averaged over a 10‐pixel height. These profiles were used to evaluate field‐edge dose fall‐off across the different dose levels. The relative dose profiles were normalized for each dose level such that the position corresponding to 50% of the maximum signal was set to *x* = 0 to facilitate comparative visualization. In addition, dose profiles were generated for the 6 MV EBT4 film strips under identical irradiation and scanning conditions to enable direct comparison with the performance of LD‐V1.

### 2.4 Scanner and film signal uniformity

An unirradiated sheet of 20 cm × 25 cm LD‐V1 Gafchromic film was cut into twenty 5 cm × 5 cm strips. Film strips were positioned across entire scanner bed with the same relative positions as in the pre‐cut film. Five back‐to‐back scans were performed, and a central 2.5 cm × 2.5 cm region‐of‐interest (ROI) was selected for each film strip using filmQA Pro.^20^ Scan‐to‐scan reproducibility was evaluated by calculating the percent difference in the mean ROI value across the five consecutive scans.^17^ Film signal uniformity was quantified as the absolute percent difference between the ROI value of each strip and the mean ROI across all strips. To assess the effect of scanner position, the film strips were translated one column and one row across the scanner so that each strip occupied a new scanner location as shown in the supplemental materials.

## 3 RESULTS

**FIGURE 1:**
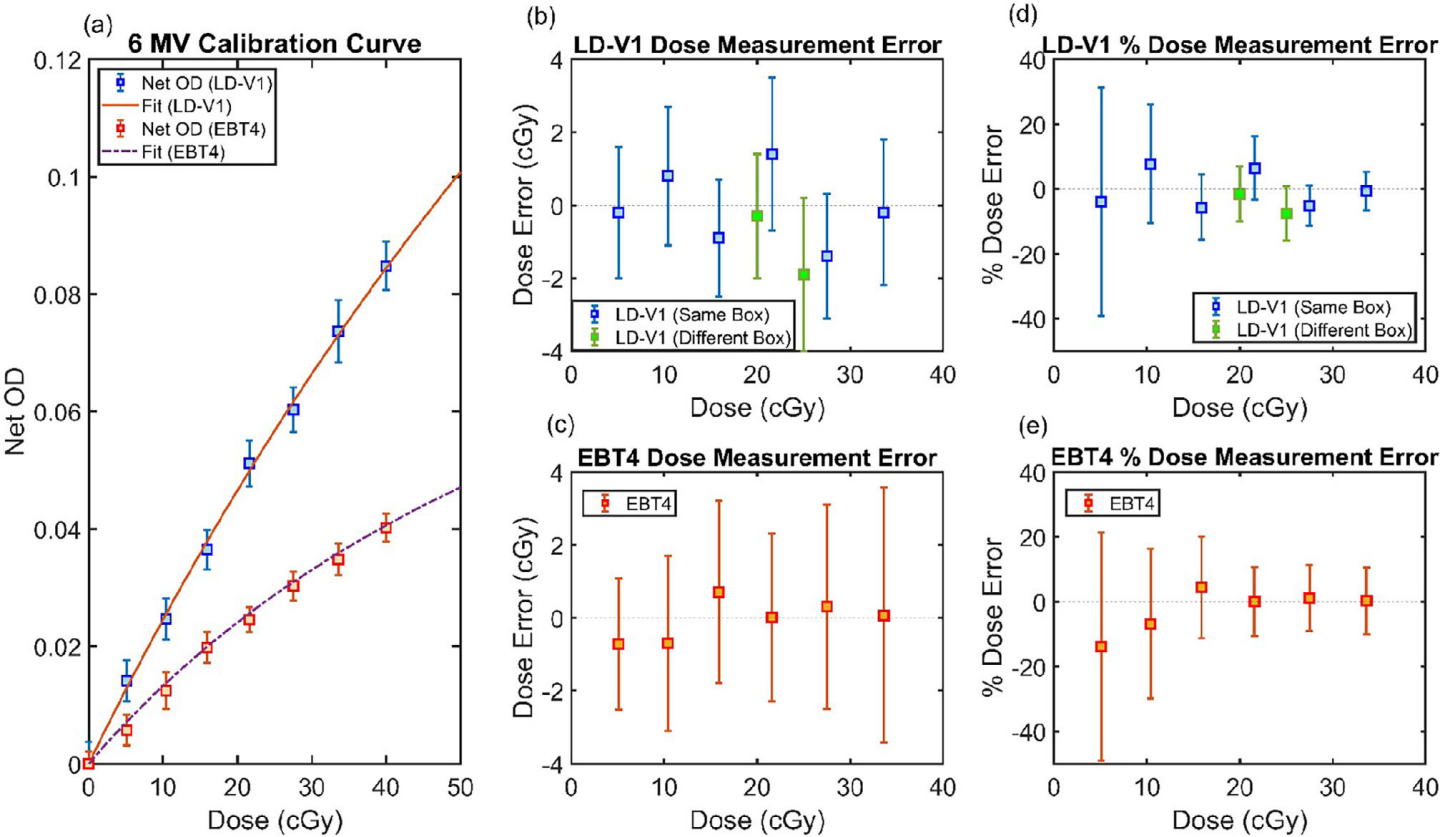
Calibration curves for LD‐V1 and EBT4 films. (a) 6 MV net optical density (OD) calibration for LD‐V1 and EBT4. (b and c) Dose measurement error (cGy) of the test films using the calibration curve for LD‐V1 and EBT4, respectively. (d and e) Relative dose error, calculated by dividing the dose measurement error to the irradiated dose for each dose level, for LD‐V1 and EBT4. All LD‐V1 films analyzed were from the same production lot. The error bars are calculated as the standard deviation of net OD (a) and dose statistics (b–e) from center 1.5 cm × 3.0 cm ROI of each film strip.

The 6 MV calibration curves in Figure 1a demonstrated a higher net OD response for LD‐V1 compared with EBT4 across all dose levels evaluated. Across all dose levels, there is no significant dose error difference between LD‐V1 and EBT4 (Figure 1b–e); however, the measurement uncertainty of LD‐V1 (1.85 ± 0.19 cGy) was significantly lower than that of EBT4 (2.55 ± 0.57 cGy; *p* = 0.017, Wilcoxon rank‐sum test). Up to 1.9 cGy dose difference was observed from measurement using LD‐V1 film from a different box within same production lot (Figure 1b).

**FIGURE 2:**
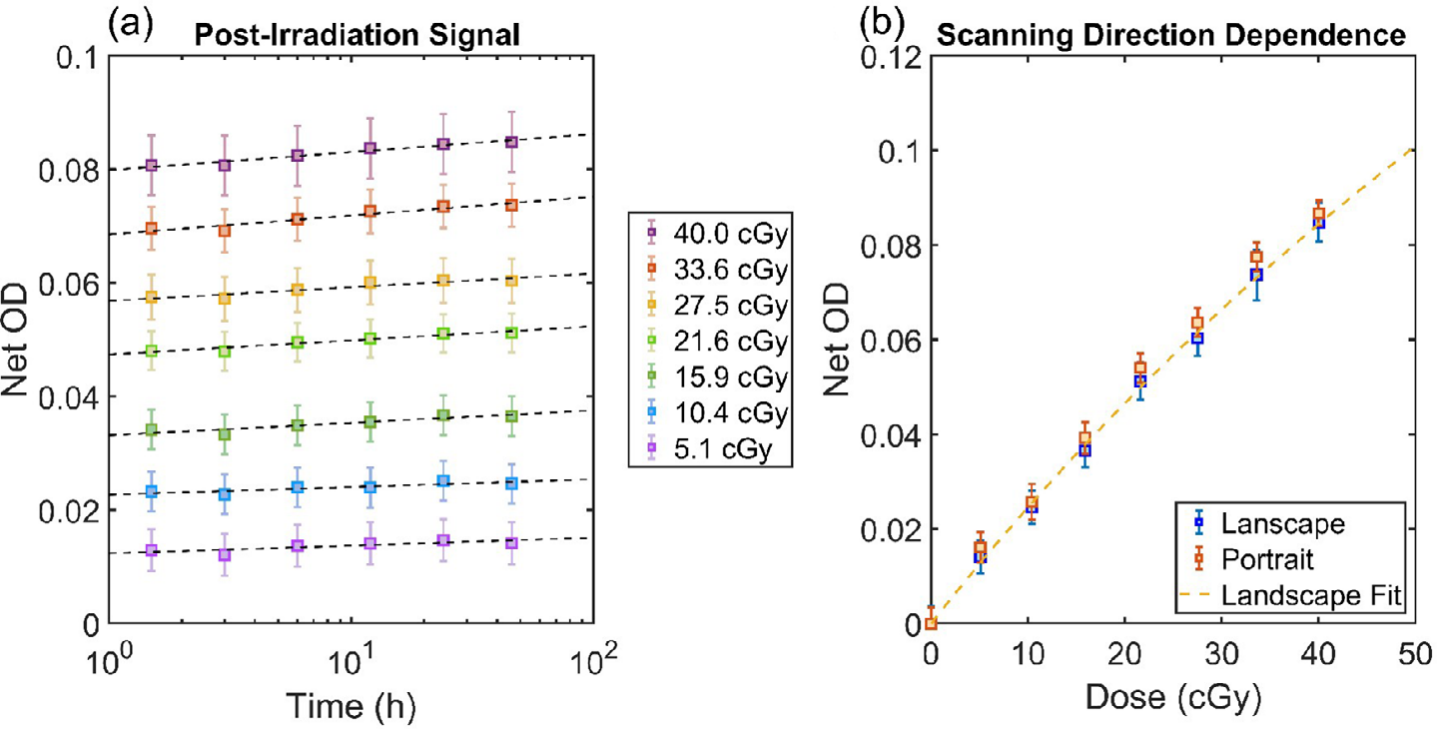
Post‐irradiation read‐out time and scanning orientation dependence of the LD‐V1 film. (a) Net optical density (OD) versus post‐irradiation time (log scale). Dashed lines represent a logarithmic fit for each dose level. (b) Net OD of film in landscape and portrait orientation in scanner bed at >24 h post‐irradiation. The error bars are calculated as the standard deviation of net OD from center 1.5 cm × 3.0 cm ROI of each film strip.

Post‐irradiation signal evolution is shown in Figure 2a. Net OD increased approximately logarithmically with time. Dose measurement error using 6 MV calibration curve shows up to 6.7% difference for the 1.5 h post‐irradiation scan, and up to 5.8% difference for the 24 h post‐irradiation scan with the implementation of one‐scan protocol of the FilmQA Pro software. Portrait‐oriented scans consistently produced higher red‐channel net OD than landscape scans, with a maximum difference of 13.8% at 5 cGy and an average difference of 6.3% across all dose levels (Figure 2b).

**FIGURE 3:**
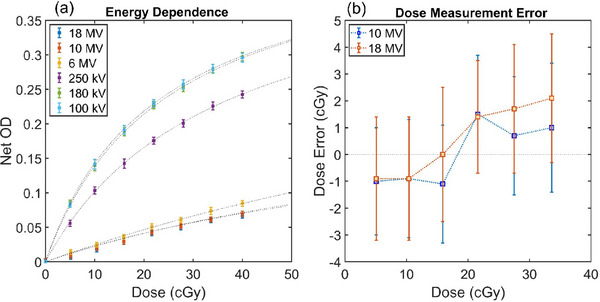
Energy dependence of the LD‐V1 film. (a) Net optical density (OD) versus dose (cGy) for 3 MV and 4 kV beam qualities. Dashed lines are calibration fit of the net OD for each energy. (b) Dose measurement errors using the 6 MV calibration curve to measure the same dose levels with 10 and 18 MV beam qualities. Error bars are calculated as the standard deviation of net OD (a) and dose statistics (b) from center 1.5 cm × 3.0 cm ROI of each film strip.

Energy‐dependence results are summarized in Figure 3. For the same delivered dose, 6 MV films produced higher net OD than films irradiated with 10 or 18 MV (Figure 3a). At 5.1 cGy, doses reconstructed using the 6 MV calibration curve underestimated the delivered dose by 19.6% and 17.6% for 10 and 18 MV films, respectively (Figure 3b). Corresponding absolute errors were 1.5 cGy for 10 MV and 2.1 cGy for 18 MV. Strong energy dependence between kV and MV energies was observed. The 250 kV beam exhibited significantly reduced sensitivity compared with the 100 and 180 kV beams at all dose levels (*p* < 0.01, Wilcoxon rank sum test^21^).

**FIGURE 4:**
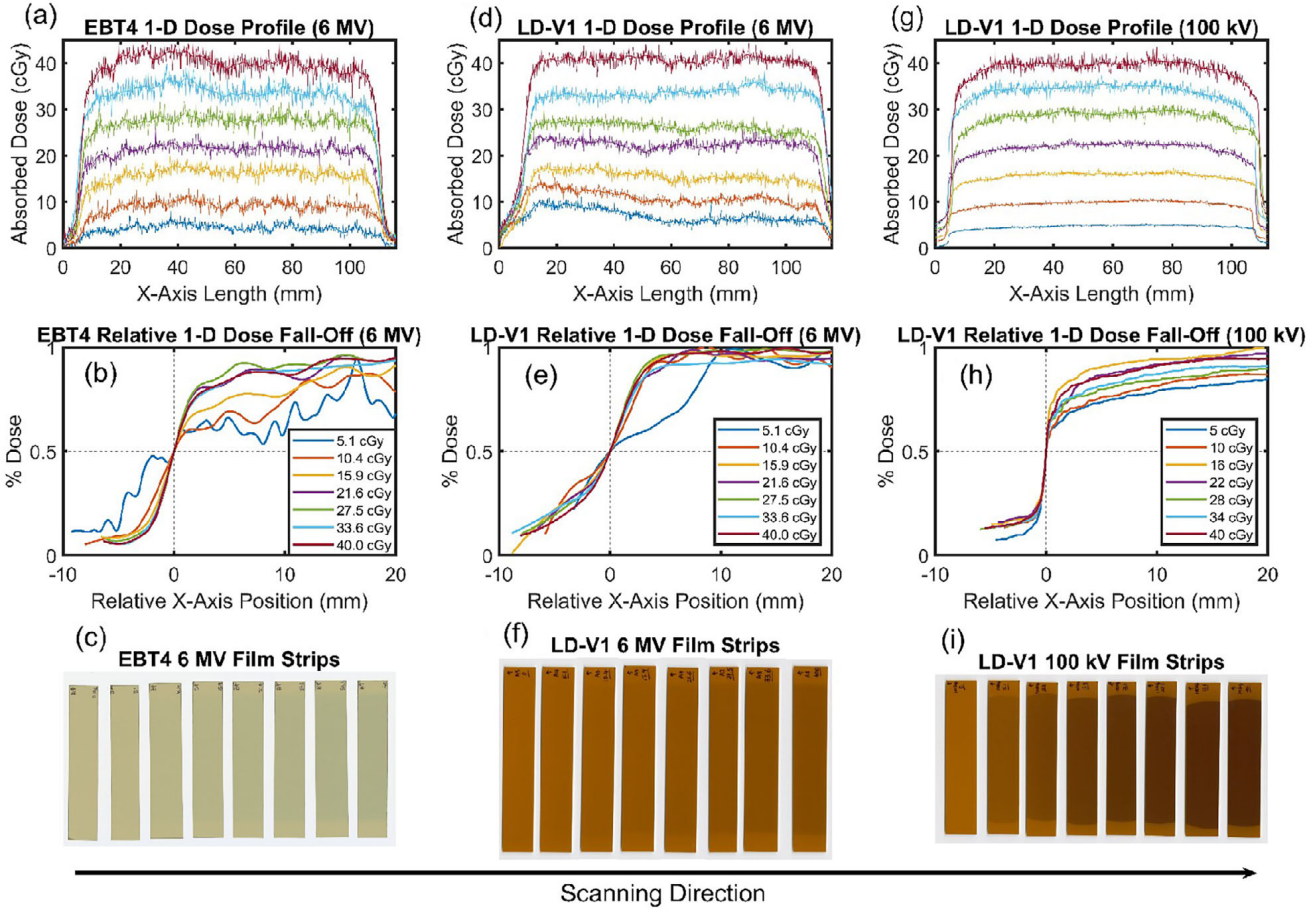
1‐D absolute and relative dose profiles for EBT4 6 MV (a, b), LD‐V1 6 MV (d, e), and LD‐V1 100 kV (g, h). The relative dose fall‐off plots are normalized to the x‐axis position (mm) where at *x* = 0 the dose reflects 50% of the max dose along each smoothed profile. Film positioning on scanner (with increased dose levels of 0 to 40 cGy from left to right) is shown in (c), (f), and (i) for 6 MV EBT4, 6 MV LD‐V1, and 100 kV LD‐V1, respectively. Horizontal profile is drawn across center of film for each dose level.

Figure 4 shows 1‐D dose profiles obtained from 6 MV and 100 kV LD‐V1 film strips, as well as the 6 MV EBT4 film strips. Field‐edge dose fall‐off was clearly visualized for all dose levels and both beam qualities. The 6 MV profiles for both films exhibit pronounced noise along the x‐axis, with the effect most evident at low doses (5.1–15.9 cGy). Under the same irradiation and scanning conditions, the LD‐V1 profiles demonstrate comparatively reduced fluctuation relative to EBT4 (Figure 4b vs. Figure 4e), reflecting its higher net OD and signal stability in the MV range.

All unirradiated film strips had <0.01% signal variation between five back‐to‐back scans, indicating excellent scan‐to‐scan reproducibility. Following translation of the film strips across the scanner bed, the maximum observed change in signal was 1.19%. Pre‐irradiation red channel intensity shows film signal uniformity variation of up to 3.87% across all film strips, with the center six strips having the highest intensities. After translation of the film on scanner (by moving the top row to the bottom and shifting all remaining rows upward), the spatial pattern of intensities remained largely unchanged for each film. This stability suggests that the measured nonuniformity arises primarily from the film sheet itself and is not attributable to spatial dependence of the scanner.

## 4 DISCUSSION

This study characterizes the low‐dose response of LD‐V1 film under MV beam conditions to assess its potential suitability for MLC QA during dynamic beam delivery. Compared with EBT4, LD‐V1 demonstrates higher net OD and significantly less absolute dose measurement uncertainty below 40 cGy, indicating improved dosimetric stability and reproducibility in the low‐dose regime. Consistent with these findings, relative dose error analysis suggests that LD‐V1 can provide improved sensitivity compared with EBT4 at doses below ∼15 cGy, where conventional MV range films often exhibit limited signal response. However, increased variability is observed for LD‐V1 at the lowest dose levels, particularly below 10 cGy, where the standard deviation of percent dose error increased substantially. This behavior is likely driven by reduced signal‐to‐noise ratio (SNR) near the lower limit of detectability. In contrast, EBT4 demonstrated superior stability within its optimal dose range with low mean dose error above 20 cGy, reflecting its design for higher‐dose MV applications. Collectively, these observations suggest that while LD‐V1 may be less suitable for precise absolute dosimetry at very low MV doses requiring sub‐cGy accuracy, its enhanced signal sensitivity and stability may still provide practical advantages for delivery pattern assessment and edge detection to verify MLC positional accuracy during highly dynamic delivery when consistent calibration and scanning protocols are applied.

LD‐V1 film exhibits considerable energy dependence between MV and kV energies as well as between 6, 10, and 18 MV energies, suggesting the need for separate calibration curves for each MV beam quality. The difference between MV and kV signal strength is likely attributable to the relatively high effective atomic number of the LD‐V1 active layer, which is optimized to enhance the photoelectric effect at low photon energies; the film exhibits a reduced dose response due to the dominance of Compton interaction for MV beams.^22^, ^23^ In comparison, EBT4 demonstrates minimal energy dependence in the MV range, with only ∼1.1% variation from 10 to 15 MV and approximately 5% reduced sensitivity at 70 kV, which is likely attributable to its more water‐equivalent effective atomic number.^24^ There is not a significant energy dependence for the LD‐V1 film response between the kV energies (100 and 180 kV), which agrees with the findings from Mbewe et al. at same energy levels irradiated on the same type of orthovoltage unit.^16^ The net OD measurements for the same dose and kV energy levels are ∼20% lower than the results from Masella et al.^17^ This discrepancy may reflect differences in setup technique and the x‐ray beam energy spectra used in the respective measurements. Although LD‐V1 contains higher‐Z components that would generally be expected to increase photoelectric interactions and produce stronger energy dependence in the kV range, this work suggests that within 100–180 kV interval the film response remains relatively stable, likely due to the balance between photon interaction processes and the active layer's polymerization mechanisms. A reduction in sensitivity at 250 kV is also observed, aligning with the manufacturer's stated low energy dependence within the 40–160 kV operating range.

Post‐irradiation signal change of the LD‐V1 film follows a logarithmic growth pattern, consistent with the observations of Masella et al.,^17^ and comparable to the post‐exposure behavior of EBT4 films.^25^ In principal, this could be addressed by using a standard readout time for all calibration and measurements (around 24 h). However, FilmQA Pro offers a “one‐scan protocol” that mathematically adjusts for post‐irradiation signal growth by use of a reference film irradiated at a known dose at approximately the same time as the film being scanned.^18^ With this protocol applied, dose measurements do not show systematic variation. Notably, films scanned in portrait orientation yield consistently higher net OD than those scanned in landscape orientation, underscoring the need for strict consistency in film orientation and scanning direction to ensure accurate dosimetry.

The comparatively smooth profile and clear visualization of field‐edge dose fall‐off of the LD‐V1 supports the film's clinical potential in MLC‐related QA such as leaf edge verifications. Quantitative measurements using the film profiles, however, require careful positioning of the film, optimized scanning resolution, lateral response artifact (LRA) corrections,^26^ and characterization of any profile‐smoothing algorithms. The 6 MV profiles exhibit noticeable signal noise along the x‐axis, similar to that reported by Masella et al,^17^ especially for low dose level of 5.1 cGy. This is consistent with the increased signal uncertainty of LD‐V1 observed below ∼16 cGy due to the decreased SNR at lower doses. The substantially lower noise observed in the 100 kV dose fall‐off region compared with the 6 MV profiles reflects the higher net OD response of LD‐V1 at kV energies, which improves SNR and reduces apparent profile fluctuations. In contrast, MV profiles operate at lower signal levels, making noise more pronounced near the field edge as the dose approaches the lower limit of detectability. Careful optimization of scanning parameters and the use of consistent acquisition protocols may help mitigate apparent profile noise in future implementations. LD‐V1 film exhibits low scan‐to‐scan variability and minimal dependence of signal on scanner‐bed position. Variation in OD (up to 3.87%) between the center and periphery of an unirradiated film was observed, compared to the manufacturer specification of 3% for EBT4 films. This variation may arise from natural curling at the film edges, which can introduce air gaps and reduce scanner contact. To reduce intra‐film variation and corresponding increase in measurement uncertainties, the films were pressed flat against the scanner surface using a glass pane,^26^, ^27^ longer film strips were used to mitigate edge curling and reduce the likelihood of edge damage and to facilitate secure gripping during handling, and films were flattened for at least 24 h prior to scanning.

There are several additional considerations when evaluating the suitability of LD‐V1 film for low dose MV QA measurements. First, LD‐V1 film was observed to gradually darken over several days under ambient room light when stored in a loosely covered envelope. The negligible signal difference between five back‐to‐back scans of unirradiated film strips suggests minimal impact scanner light on LD‐V1's signal response; however, careful storage of the film in darkened conditions is still recommended prior to scanning to avoid unintended signal changes from ambient light exposure. In addition, because LD‐V1 is optimized for low‐dose measurements, the film requires meticulous handling to avoid scratches, fingerprints, and other mechanical damage that could compromise dosimetric accuracy. First, only red channel intensity is used for all calibration, fitting, and dose calculation due to its higher sensitivity and precision determined from previous findings.^17^ Alternatively, studies have shown that triple channel dosimetry could improve the dose measurement accuracy by reducing impact of dose‐independent effects such as film non‐uniformities and scanner‐dependent properties.^28^ A comparison between single and multi‐channel dose determination could be of interest. Third, scanner characteristics, such as scanning resolution and LRA, could have a considerable impact on the dosimetric accuracy, especially that of the 1‐D dose profile. While recent LD‐V1 studies have used 72–150 dpi scanning resolution,^13^, ^14^, ^15^, ^16^, ^17^ a higher resolution of 200 dpi was selected in this study to improve spatial delineation of field‐edge fall‐off. This choice reflects the spatial demands for the film's intended clinical applications (e.g., MLC QA) rather than signal optimization. Although higher resolution may sharpen the measured dose profile, it can also increase noise in the dose profile, leading to reduced SNR. Relatively large uncertainty (∼2 cGy) in both EBT4 and LD‐V1 measurements were observed, which is consistent with higher sampling noise at increased scan resolution: if noise is dominated by pixel‐level fluctuations, the standard deviation scales approximately with the square root of the number of pixels per unit distance, that is, by √(200/72) ≈ 1.67 relative to 72 dpi. Such trade‐offs between scanning resolution and SNR warrant further systematic investigation in future work. A commonly used strategy to improve SNR performance of EBT4 films in low‐dose regions is repeated irradiation followed by normalization to the number of exposures during post‐processing. However, the primary motivation for this study is to develop a tool to evaluate position reproducibility, which could be obscured by repeat measurements. Furthermore, repeated exposures increase beam time, introduce additional setup uncertainty, and may not be practical for routine or high‐throughput clinical QA workflows. In contrast, the higher intrinsic low‐dose response of LD‐V1 may reduce the need for such signal‐amplification strategies when performing single‐exposure MV measurements. With careful optimization of scanning and analysis protocols, such as the addition of LRA corrections, LD‐V1 could achieve further improved dosimetric accuracy, allowing for potential applications beyond MLC QA, including in‐vivo peripheral dose monitoring to pregnant patients or patients with cardiac implantable electronic devices.

## 5 CONCLUSIONS

LD‐V1 demonstrates strong potential for low‐dose MV dosimetry, outperforming EBT4 in signal strength and dosimetric uncertainty for doses below 40 cGy. The film exhibits pronounced energy dependence across both MV and kV beam qualities, indicating that a dedicated calibration curve is required for each MV energy. LD‐V1 also shows substantial sensitivity to scanning orientation, underscoring the need for consistent alignment between calibration and measurement. A minimum readout time of 24 h post‐irradiation is recommended for signal stability, although the one‐scan protocol may be used when earlier scanning is necessary. Intra‐film variation is observed at low dose, and flattening the film prior to scanning may help mitigate associated uncertainties. Finally, the clear visualization of field‐edge dose fall‐off in the 1‐D profiles suggests that LD‐V1 may have valuable applications in MLC QA, such as leaf positioning verifications. While the increased noise at the lowest dose levels may limit precise quantitative dosimetry at sub‐cGy accuracy, the film continues to provide meaningful relative dosimetric information and may achieve improved quantitative performance with further optimization of scanning and analysis protocols. Future work will include 1‐D and 2‐D profile characterization under rapid MLC, collimator, and gantry motion with to assess the film's potential roles in advanced clinical QA implementations.

## AUTHOR CONTRIBUTIONS

**Ruiming Edmondson**: conception and design, data collection, data analysis and manuscript writing, edits and final approval of manuscript. **Darwin Garcia**: data collection, edits and final approval of manuscript. **Victor Malkov**: conception and design, edits and final approval of manuscript. **James Kavanaugh**: conception and design, edits and final approval of manuscript. **John Lucido**: conception and design, edits and final approval of manuscript.

## CONFLICT OF INTEREST STATEMENT

The authors have no relevant conflicts of interest to disclose.

## Supporting information



Supporting File 1: acm270578‐sup‐0001‐SuppMat.jpg

Supporting File 2: acm270578‐sup‐0002‐SuppMat.docx

## Data Availability

Research data are stored in an institutional repository and will be shared upon request to the corresponding author.
