# Supplementary figures and images for: Assessment of LD‐V1 radiochromic film low‐dose performance for mega‐voltage radiotherapy quality assurance
Source: J Appl Clin Med Phys. 2026 Apr 9;27(4):e70578. doi: 10.1002/acm2.70578 (PMC13065876; doi:10.1002/acm2.70578)

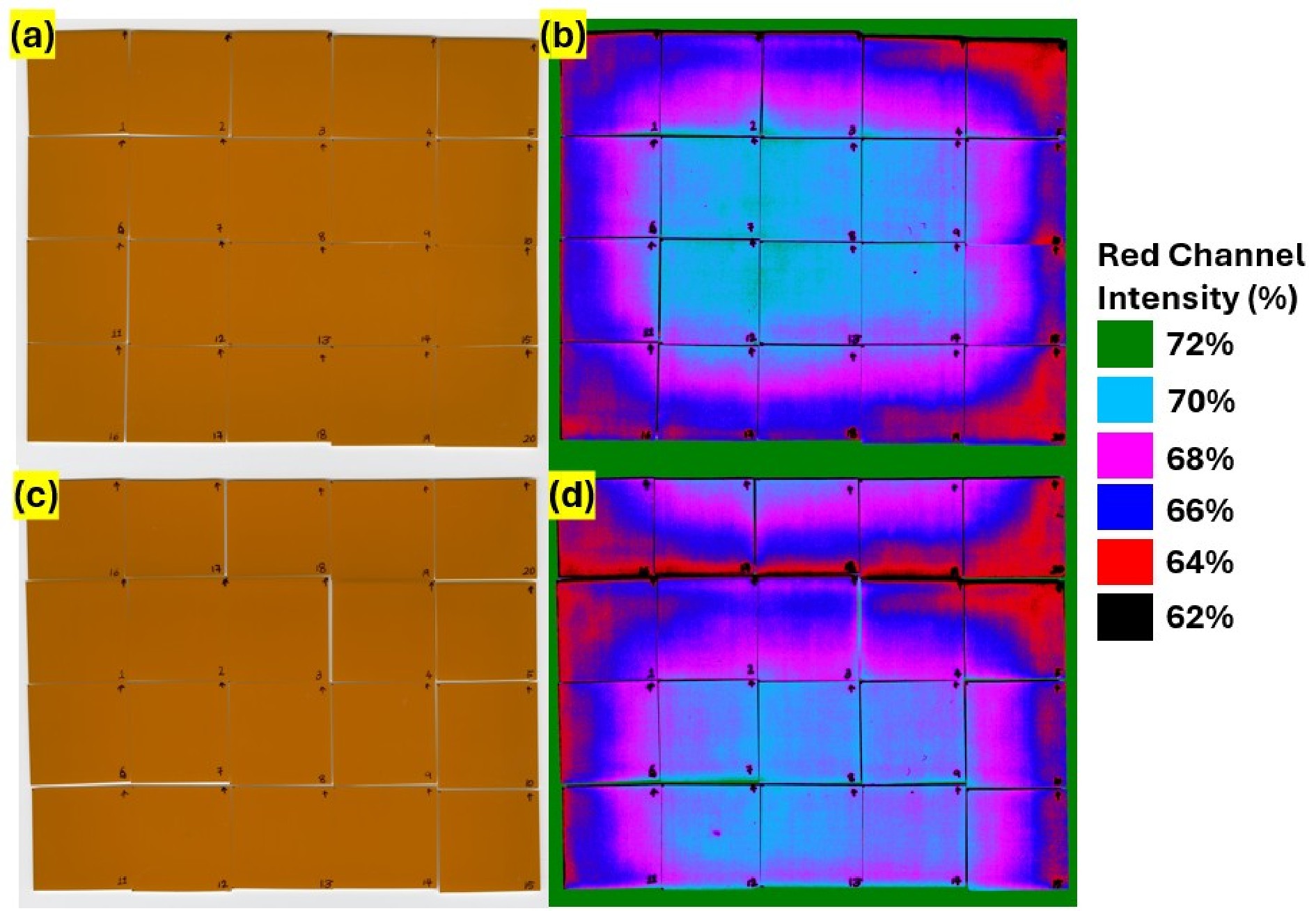

Supplement: Supplementary file 1 — Supporting File 1: acm270578‐sup‐0001‐SuppMat.jpg [file ACM2-27-e70578-s001.jpg]
