# Supplementary material for: Assessment of LD‐V1 radiochromic film low‐dose performance for mega‐voltage radiotherapy quality assurance
Source: J Appl Clin Med Phys. 2026 Apr 9;27(4):e70578. doi: 10.1002/acm2.70578 (PMC13065876; doi:10.1002/acm2.70578)
Supplement: Supplementary file 2 — Supporting File 2: acm270578‐sup‐0002‐SuppMat.docx [file ACM2-27-e70578-s002.docx]

Film and scanner uniformity characterization. Pre-irradiation scan and red channel intensity of the cut film strips in their original positions in film is shown in a) and b). Red channel intensity is calculated as the percentage of raw red channel pixel value over the 16-bit flood field value of 65535. After translation of the film on scanner (by moving the top row to the bottom and shifting all remaining rows upward, c and d), the spatial pattern of intensities remained largely unchanged for each film. This stability suggests that the measured nonuniformity arises primarily from the film sheet itself and is not attributable to spatial dependence of the scanner.
